# Identifying biomarkers of breast cancer micrometastatic disease in bone marrow using a patient-derived xenograft mouse model

**DOI:** 10.1186/s13058-017-0927-1

**Published:** 2018-01-02

**Authors:** Sreeraj G. Pillai, Shunqiang Li, Chidananda M. Siddappa, Matthew J Ellis, Mark A. Watson, Rebecca Aft

**Affiliations:** 1 Department of Surgery, Washington University School of Medicine, 660 South Euclid Avenue, St. Louis, MO 63110 USA; 2 Department of Internal Medicine, Division of Medical Oncology, Washington University School of Medicine, St. Louis, MO USA; 3 Baylor College of Medicine, Lester and Sue Smith Breast Center, Houston, TX USA; 4 Department of Pathology and Immunology, Washington University School of Medicine, St. Louis, MO USA; 5 Siteman Cancer Center at the Washington University School of Medicine, St. Louis, MO USA; 6 John Cochran Veterans Administration Hospital, St. Louis, MO USA

**Keywords:** Breast cancer, Disseminated tumor cells, Patient-derived xenograft, Metastasis, Gene expression profile

## Abstract

**Background:**

Disseminated tumor cells (DTCs) found in the bone marrow (BM) of patients with breast cancer portend a poor prognosis and are thought to be intermediaries in the metastatic process. To assess the clinical relevance of a mouse model for identifying possible prognostic and predictive biomarkers of these cells, we have employed patient-derived xenografts (PDX) for propagating and molecularly profiling human DTCs.

**Methods:**

Previously developed mouse xenografts from five breast cancer patients were further passaged by implantation into NOD/SCID mouse mammary fat pads. BM was collected from long bones at early, serial passages and analyzed for human-specific gene expression by qRT-PCR as a surrogate biomarker for the detection of DTCs. Microarray-based gene expression analyses were performed to compare expression profiles between primary xenografts, solid metastasis, and populations of BM DTCs. Differential patterns of gene expression were then compared to previously generated microarray data from primary human BM aspirates from patients with breast cancer and healthy volunteers.

**Results:**

Human-specific gene expression of *SNAI1, GSC, FOXC2, KRT19,* and *STAM2*, presumably originating from DTCs, was detected in the BM of all xenograft mice that also developed metastatic tumors. Human-specific gene expression was undetectable in the BM of those xenograft lines with no evidence of distant metastases and in non-transplanted control mice. Comparative gene expression analysis of BM DTCs versus the primary tumor of one mouse line identified multiple gene transcripts associated with epithelial-mesenchymal transition, aggressive clinical phenotype, and metastatic disease development. Sixteen of the PDX BM associated genes also demonstrated a statistically significant difference in expression in the BM of healthy volunteers versus the BM of breast cancer patients with distant metastatic disease.

**Conclusion:**

Unique and reproducible patterns of differential gene expression can be identified that presumably originate from BM DTCs in mouse PDX lines. Several of these identified genes are also detected in the BM of patients with breast cancer who develop early metastases, which suggests that they may be clinically relevant biomarkers. The PDX model may also provide a clinically relevant system for analyzing and targeting these intermediaries of metastases.

**Electronic supplementary material:**

The online version of this article (doi:10.1186/s13058-017-0927-1) contains supplementary material, which is available to authorized users.

## Background

Multiple prospective clinical trials have demonstrated that disseminated tumor cells (DTCs) found in the bone marrow (BM) of patients with early-stage breast cancer are highly correlated with early recurrent disease development and portend a poor prognosis [1, 2], even many years after initial diagnosis [3]. BM DTCs are thought to be intermediaries in the metastatic process, transitioning in the BM, re-entering the circulation, and proliferating in distant organs with a favorable molecular micro-environment [4]. DTCs in the BM may be indicative of the systemic burden of micrometastatic disease in the patient [2]. Those patients with residual DTCs after chemotherapy are at very high risk of recurrence, indicating that those cells that survive chemotherapy have high metastatic potential [5]. Recent animal models suggest that early disseminated cells evolve in parallel to the primary tumor and have high metastatic potential [6, 7]. To prevent the development of metastatic outgrowth, it is necessary to devise therapeutic strategies to target the intermediary cancer cells that evade conventional treatment.

To date, primary DTCs have been difficult to characterize. The rarity of these cells, the lack of uniform markers for detecting cells with metastatic potential, and the evolution of the cells while in a foreign micro-environment are the main constraints in identifying, isolating, and molecularly characterizing DTCs from patient BM specimens [8]. To address these limitations, we have investigated the use of patient-derived xenograft mouse models (**PDX**), wherein primary human breast carcinomas are transplanted and propagated in the mammary fat pad of mice, as a continuous, reproducible source of disseminated tumor cells for molecular characterization. Multiple studies have documented that the molecular profile, histopathological characteristics, and therapeutic sensitivities of PDX tumors recapitulate that of their primary tumor counterparts, and therefore should serve as an excellent model for tracking, studying, and testing interventions for metastatic disease development [9–11]. Evidence from other studies shows that primary, peripheral blood, circulating tumor cells (CTCs) from patients with breast cancer can also survive and propagate as mouse xenografts, again suggesting phenotypic parallels between PDX and human metastases [12, 13]. Recently, detection of CTCs and DTCs has been reported in a PDX model [14].

In this report, using a PDX system established by transplanting primary tumors from pre-metastatic patients with breast cancer, we demonstrate that development of distant organ metastases correlates with the presence of BM DTCs. Comparative gene expression analysis of BM from these animals has allowed the identification of novel gene expression patterns associated with DTC colonization of BM and further supports the concept that DTCs present in the BM undergo epithelial to mesenchymal transition (EMT). Moreover, the expression of many of the genes identified in this PDX model distinguish BM from patients with breast cancer who develop early metastatic relapse from that of healthy female volunteers, suggesting potential value as prognostic and predictive biomarkers. We believe that the PDX model is an effective tool to identify and study the molecular characteristics of BM DTCs and their role in the metastatic process, and should allow for the development of new therapies to target these cells.

## Methods

### Patient population and establishment of PDX lines

After patients gave informed consent, human breast adenocarcinomas were prospectively collected using a protocol approved by the Institutional Review Board at Washington University in St. Louis, and transplanted into mice. All animal procedures were reviewed and approved by the Institutional Animal Care and Use Committee at Washington University in St. Louis. Briefly, after 3-week old female NOD-SCID mice were anesthetized, an inverted Y-shaped incision was made to expose the mammary glands. Using a dissecting microscope, the lymph-node and the vessel in the fat bridge between the fourth and fifth mammary fat pads were cauterized. The breast epithelium in this area was then excised to create the “cleared fat pad” into which human breast tissues were implanted without interference from the host’s mammary epithelium. At 2 weeks post clearance, 500,000 immortalized green fluorescent protein (GFP)-labeled human breast fibroblasts were injected into each cleared fat pad. After an additional 2 weeks, the humanized fat pads received tumor implants. Breast biopsies were prepared for engraftment by placing tissue in ice-chilled high glucose DMEM, immediately transporting it to the laboratory, and mincing into 1–2-mm pieces for implantation in up to five mice. Further details of the development and maintenance of xenograft lines has been previously described [9–11]. Individual animals are designated using the labeling convention: primary tumor line *–* passage – unique animal ID (e.g. 17-B-1141). Clinical features and pathological characteristics of the five patient tumor xenograft lines that were used for the present studies are listed in Table 1.

**Table 1:** Clinical details and pathological characteristics of tumor specimens used for generating the PDX WHIM lines, as determined and further described in a previous publication [11]

		Patient details				Xenograft details			
Pt ID	Receptor status	Subtype	Source of tissue	Sites of metastasis	Survival (months)	WHIM line	Mouse ID	Receptor status	Site of metastases
262	ER+/HER2-	Luminal-B	Breast	BN, CNS	37	11	11E 1221	ER+/HER2-	None
303	ER-/HER2-	Claudin-low	Breast	PL, PC	12	12	12A 1282	ER-/HER2-	L
							12A1291		L
							12C1363	ER-/HER2-	H
							12C1264		None
							12C 1375		None
							12C 1376		None
270	ER-/HER2-	Basal	Chest wall	BR, CT, L, H, BN	44+ ^b^	13	13A 1204	ER-/PR-	None
7192	ER-/HER2-	Claudin-low	Breast	None	37+ ^b^	17	17A 1384	ER-/HER2-	H, N
							17B 1139	ER-/HER2-	H
							17B 1141		L
							17B1188		L, H
							17B29		L, H, S
							17C1180	ER-/HER2-	L
							17C1182		L, H, S, K
319	ER+/HER2-	Luminal-B	Skin	CT, N	97	23	23C 1832		None
							23C 1833	ER+/HER2-	None
							23C 1834		None

Individual mice used for bone marrow analysis are designated by: WHIM line number - passage number – unique mouse ID

*Abbreviations: PDX* patient-derived xenograft, *WHIM* Washington University Human in Mouse *ER* estrogen receptor, *HER2* human epidermal growth factor receptor 2, *BR* breast, *CNS* brain, *BN* bone, *L* lung, *CT* cutaneous, *PL* pleura, *PC* pericardium, *N* nodes, *H* liver, *S* spleen, *K* kidney, *PR* progesterone receptor

^b^Alive at last follow up

### BM and RNA isolation

Mice were killed when the primary xenograft tumor reached approximately 1.5 cm in size (approximately 6–8 weeks after implanting the tumor tissues). The femur and tibia were dissected from surrounding tissue, avoiding potential contamination, and flushed with cold PBS to isolate BM cells. Normal mouse BM samples were collected from non-tumor-bearing NOD-SCID mice, both with and without transplanted human fibroblasts. BM from the four long bones of each animal was pooled and cells pelleted for RNA extraction. Total RNA was isolated from samples using Trizol reagent (Invitrogen) according to manufacturer’s protocol. The extracted RNA was quantified and qualitatively assessed using an Agilent Bioanalyzer.

### qRT-PCR

One microgram of RNA was used for synthesis of first-strand complementary DNA (cDNA) using the Retroscript (Ambion) kit with random hexamers. Resulting cDNA was diluted to an equivalent of 10 ng/μL of input RNA. qRT-PCR of the indicated genes was performed as described previously [8]. Human specific primer/probe sets for the genes tested were purchased from Applied Biosystems and the assay ids of the probes used are given in Additional file 1: Table S1. Each reaction consisted of 2 μL of cDNA, TaqMan Master Mix (Applied Biosystems) and primer/probe set in a total volume of 20 μL. For each transcript/sample, triplicate reactions were run in an ABI 7500 FAST Sequence Detection System. If a transcript was not detected in at least two replicates by cycle 40, it was considered absent in that sample and excluded from further analysis. Reactions with a cycle threshold (*C*_T_) value difference >1.5 for the same probe were also excluded from further analysis. The *C*_T_ values of each gene were normalized to mouse glyceraldehyde-3-phosphate dehydrogenase (GAPDH) *C*_T_ values for the same sample. These delta C_T_ (dC_T_) values were then normalized to corresponding dC_T_ values of the non-tumor-bearing mice for the same transcript and fold change calculated using the dd*C*_T_ method. Transcripts that did not reach C_T_ in non-tumor-bearing control mouse samples after 40 cycles were assigned a C_T_ value of 40 for calculation purposes.

### Microarray analysis

**Fig. 1:**
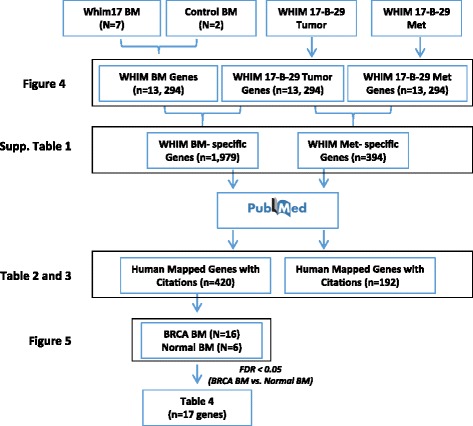
Data sets, analyses, and workflows utilized for identifying disseminated tumor cell gene expression biomarkers. *BM* bone marrow, *Met* metastasis, *BRCA* breast cancer, *WHIM* Washington University Human in Mouse

Gene expression profiling was performed as previously described [8]. Total RNA was used for two-cycle biotinylated cRNA target synthesis (Affymetrix). Resulting biotinylated cRNA was quantified and samples that yielded >15 μg of cRNA were used for GeneChip microarray hybridization. Fragmented, biotinylated cRNAs were hybridized to Affymetrix Human Gene 1.0 ST microarrays following standard protocols. Arrays were hybridized, washed, and scanned following the manufacturer's protocol. GeneChip CEL files were processed with the RMA algorithm and normalized using Partek Genomics Suite software. Differential patterns of gene expression were identified from annotated, normalized microarray data as detailed in the “Results” section. All data filtering, visualization, and analysis of variance (ANOVA) was performed using Partek Genomics Suite software. A schematic of data sets utilized and analysis workflows are presented in Fig. 1. Gene expression data are available at Gene Expression Omnibus [GEA:GSE57947].

## Results

### Development of metastatic tumors correlates with presence of human cells in mouse BM

To investigate the clinical relevance of PDX models for studying BM DTCs in patients with breast cancer, we utilized a set of previously characterized PDX mouse lines [9, 11, 15]. BM was collected from a total of 18 animals, spanning five different passages and representing initial implants from five different patients with a variety of molecular phenotypes (Table 1). All but one patient (7192) developed distant, clinical metastatic disease.

**Fig. 2:**
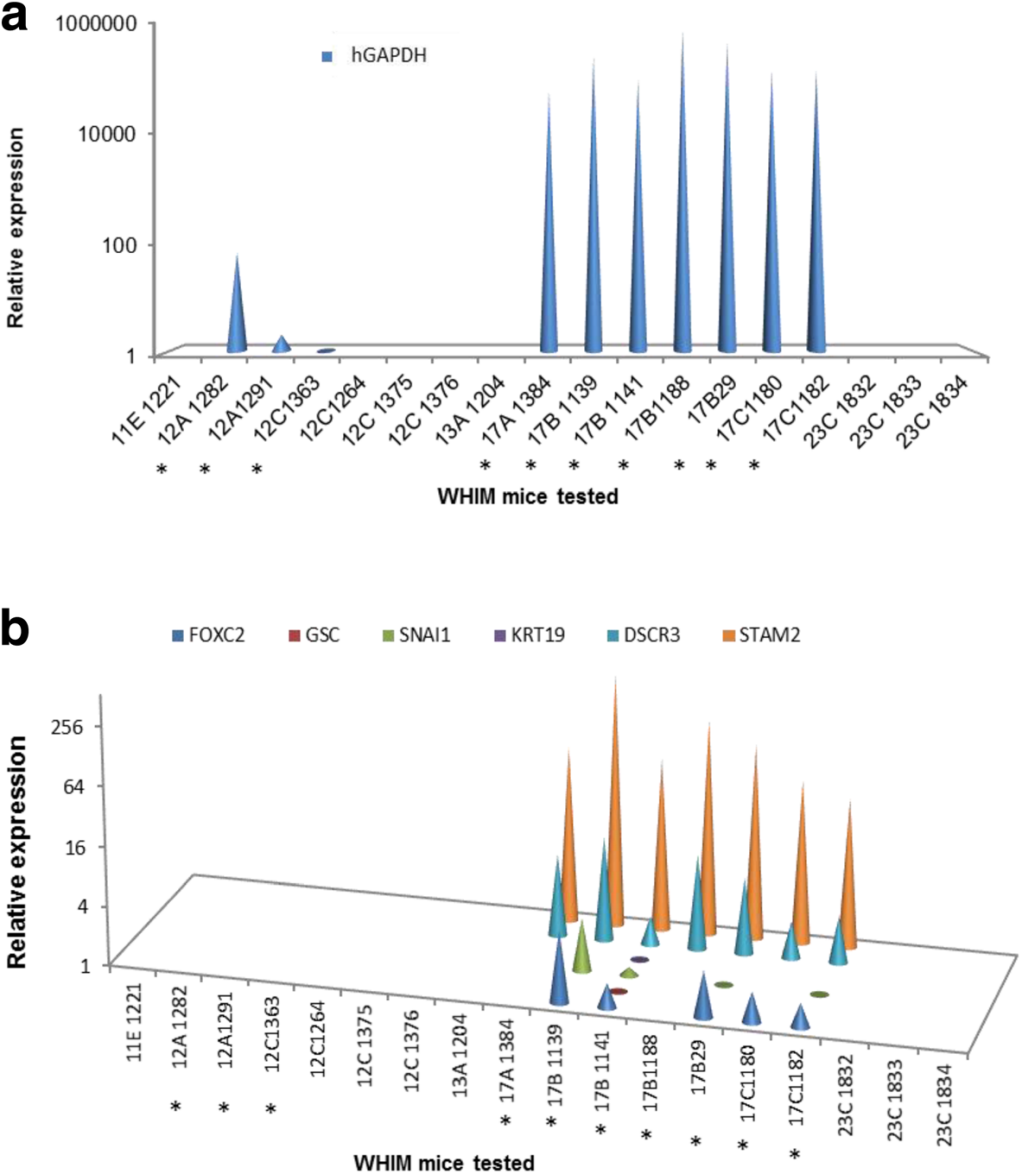
Expression of human-specific *GAPDH (hGAPDH)* (**a**) and other biomarker genes of epithelial cell lineage and epithelial to mesenchymal transition (**b**), detected in the bone marrow (BM) of patient-derived xenograft (PDX) mice. Expression of each transcript in the BM of tumor bearing mice is represented relative to that in non-tumor-bearing humanized NOD-SCID mice (control), using the dd C_T_ method. Since human-specific transcripts were not detected in the BM of control mice, for calculation purposes, a C_T_ value of 40 was assigned. *Animals that developed metastatic tumors. The association between metastatic outcome and gene expression was statistically significant for *hGAPDH* (*p* < 0.0001), *STAM2* (*p* = 0.004), *DSCR3* (*p* = 0.004) and FOXC2 (*p* = 0.036) analyzed by the Fisher exact test

To allow for multiple molecular analyses with limited amounts of BM, we employed a molecular screen to detect BM DTCs in each animal, based on detection of human-specific GAPDH (*hGAPDH*) transcript. As shown in Fig. 2a, 10 of 18 (55%) animals analyzed had detectable expression of *hGAPDH* in their BM. *hGAPDH* expression clearly emanated from BM DTCs as other humanized xenograft animals and non-grafted controls, but fat pad humanized animals, had no detectable expression of *hGAPDH* (data not shown). Furthermore, all BM samples were assayed for *GFP* gene expression and found to be negative (data not shown), suggesting that *hGAPDH* expression emanated from actual DTCs and not from the GFP-labelled human fibroblasts that were implanted and that may have migrated from fat pad implantation. Although the actual number of human DTCs present in the BM of each mouse could not be calculated based on qRT-PCR data, assuming that *hGAPDH* expression levels per input mass of total RNA are proportional to DTC cell numbers, it is clear that WHIM17 mice maintained a much higher tumor burden in their BM, as compared to those from the WHIM12 line (Fig. 2a).

Among the DTC-positive mice, seven originated from the WHIM17 line and three originated from the WHIM12 line. These ten animals all developed distant solid metastases, primarily to the lung and liver (Table 1). In contrast, eight other animals, originating from lines WHIM11, WHIM12, WHIM13, and WHIM23 had neither detectable expression of *hGAPDH* in their BM nor any evidence of distant solid metastases, even after primary tumor growth had progressed to 1.5 cm at their greatest diameter at the time of sacrifice. The presence of BM DTCs and the development of distant metastases were highly correlated (*p* < 0.0001, Fisher’s exact test), consistent with clinical observations of DTCs and metastatic disease development in patients [1, 8]. In fact, although only 18 animals were analyzed in this study, detection of *hGAPDH* expression in BM was 100% specific and 100% sensitive for predicting metastatic spread of the xenograft tumor.

### Human DTCs in mouse BM express markers of epithelial-mesenchymal transition (EMT)

Data suggest that only those cancer cells that undergo extensive molecular and phenotypic adaptations, such as EMT, will successfully survive and proliferate in a foreign micro-environment [16]. We therefore examined the BM of the PDX mice for the expression of genes associated with both epithelial cell lineage and EMT using directed, qRT-PCR analyses for human-specific gene expression. EMT-associated transcripts included Snail1 (*SNAI1*), Gooscoid (*GSC*), and *FOXC2*. As expected, in control and non-metastatic mice without *hGAPDH* expression in BM, none of the epithelial and EMT marker genes were detected. In eight of the ten *hGAPDH*-positive mice, epithelial marker genes often used for DTC detection in human studies, i.e keratin17 (*KRT17*), mammaglobin (*SCGB2A2*), and EpCAM (*TACSTD1*) were also not detected. Keratin19 *(KRT19*) expression was detected in only one animal derived from the WHIM17 line (i.e. 17-B-1141). In contrast, expression of three EMT marker transcripts, Snail1 (*SNAI1*), Gooscoid (*GSC*), and *FOXC2* were detected in many, albeit not all, of the seven WHIM17-derived animals (Fig. 2b) but in none of the WHIM12 mice. Since *hGAPDH* expression in the WHIM12 animals was also lower, this may simply reflect lower tumor (DTC) burden in the BM of these animals.

### Comparative molecular profiles of DTCs and their corresponding primary and metastatic tumors

To better understand the molecular evolution of tumor metastasis we utilized one PDX line (WHIM17) to compare patterns of gene expression between primary xenograft tumor, BM DTC populations, and distant solid organ metastasis. Of specific interest were patterns of gene expression that were unique to DTC populations, and groups of genes that were common to both DTCs and solid organ metastasis, but distinct from those of the primary tumor itself.

**Fig 3:**
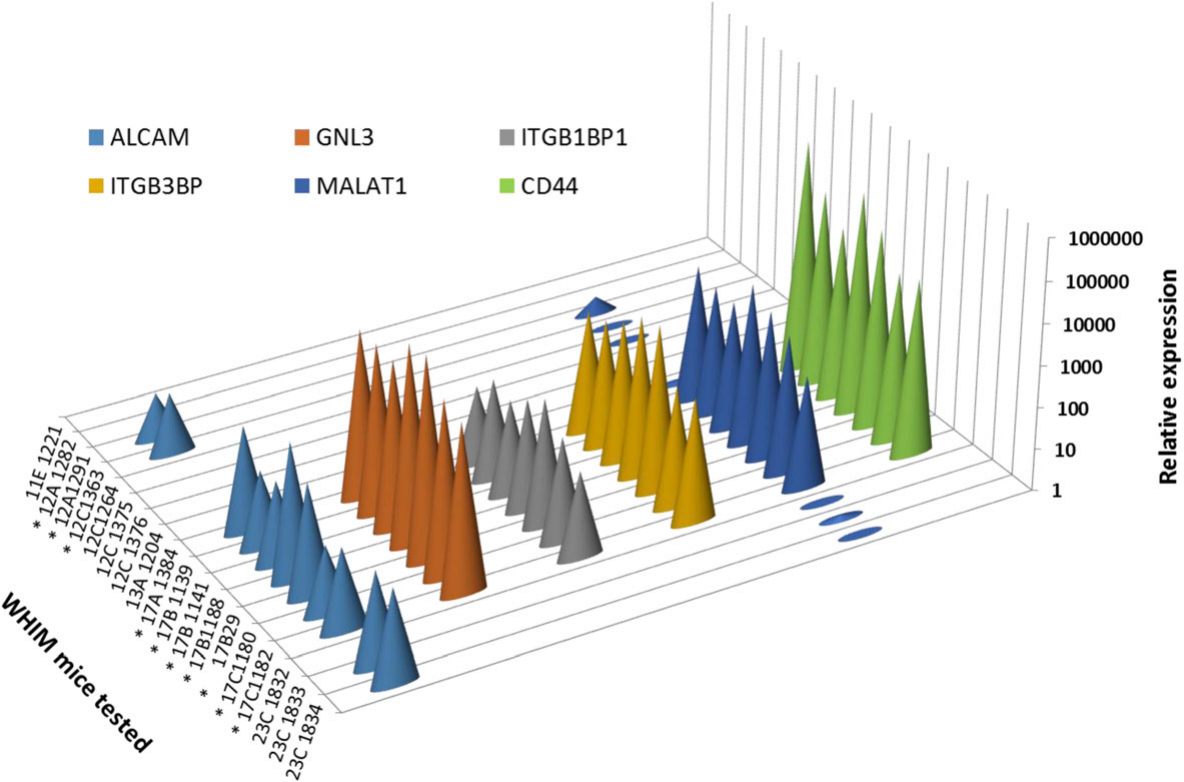
Validation of expressed genes identified from microarray analysis of Washington University Human in Mouse (WHIM)17 bone marrow (BM) samples, by qRT-PCR. Expression of each transcript in the BM of tumor-bearing mice is represented relative to that in non-tumor-bearing humanized NOD-SCID mice (control), using the dd C_T_ method. Since human-specific transcripts were not detected in the BM of control mice, for calculation purposes, a C_T_ value of 40 was assigned. *Animals that developed metastatic tumors. The association between metastatic outcome and gene expression was statistically significant for *ALCAM* (*p* = 0.013), *GNL3* (*p* = 0.004), *ITGB1BP1* (*p* = 0.004). *ITGB3BP* (*p* = 0.004), *MALAT1* (*p* = 0.023 and *CD44* (*p* = 0.004) analyzed by the Fisher exact test

Gene expression microarray analysis was performed on the WHIM17 primary xenograft tumor (17-B-29), a splenic metastasis that developed in that animal (17-B-29), and one DTC-positive BM sample from the same animal and six from different passages from the same line. Although human-specific microarrays were used for this analysis, it was expected that some patterns of gene expression in the mouse BM samples could originate from cross-hybridization of transcripts from murine BM cells. Therefore, we also profiled two BM samples from both control mice and non-engrafted mice with humanized mammary fat pads. Expression data from these animals was used as a baseline to identify human DTC-specific expression in the BM of each of the WHIM17 animals. To validate this *in silico* approach, we selected six transcripts of genes previously implicated in tumorigenesis and metastasis [17–26] the expression of which was elevated at least threefold in all seven WHIM BM samples, as compared to control BM, and confirmed expression using human-specific primers and qRT-PCR (Fig. 3, Additional file 2: Table S2).

*GLN3*, *ITGB3BP*, *MALAT1*, and *ITGB1BP1* were detected in the BM specimens from all WHIM17-derived mice, but not in the BM of mice derived from any other line. *CD44* and *ALCAM* expression was detected in both mice that developed (WHIM17 and WHIM12) and mice that did not develop (WHIM23) metastatic disease. None of the non-tumor-bearing control mice with humanized mammary fat pads demonstrated expression of any of these genes (Fig. 3).

**Fig. 4:**
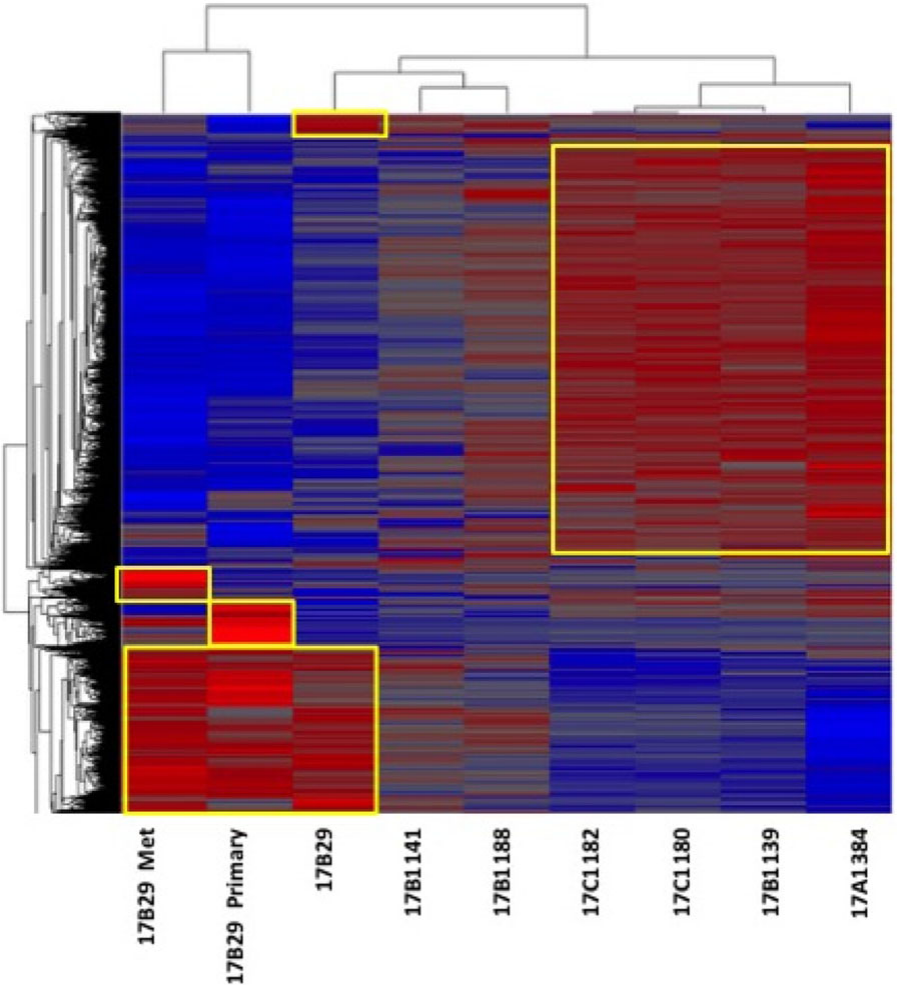
Unsupervised hierarchical cluster analysis of 13,294 gene transcripts across seven samples of Washington University Human in Mouse (WHIM17) bone marrow (BM) and corresponding primary tumor and metastatic lesion. Specific sample numbers are indicated (see Table 1) and gene expression clusters specifically upregulated in primary tumor, metastasis, and a subset of BM samples are highlighted

As shown in Fig. 4, global gene expression analysis of seven WHIM17 BM samples and corresponding tumor and metastatic lesion from the WHIM 17B29 animal showed specific clusters of genes with upregulated expression in both the primary tumor and the metastatic lesion. Surprisingly, there was considerable variability in gene expression among BM samples from the WHIM17 animals that appeared independent of tumor passage, pattern of metastatic spread, RNA quality, or other technical parameters. Not surprisingly, the WHIM17B29 BM expressed the greatest resemblance to the primary and metastatic lesion from the same animal, while four other BM samples shared a unique profile with a large number of transcripts that were over represented compared to primary tumor, metastasis, or other *hGAPDH*-positive BM samples.

We focused on clusters of 1979 unique “BM-specific” and 394 unique “metastasis-specific” transcripts to identify those that may be most relevant as biomarkers for the presence of DTCs. Additional file 3: Table S3 provides a complete list of those transcripts with significantly different expression between DTCs and primary tumors, and metastasis and primary tumor, while Tables 2 and 3 provide further filtered lists of those transcripts most highly associated with “metastasis” in the published literature in a comparison between metastases versus primary tumor and BM versus primary tumor. Many of these genes could be therapeutically targeted and are at different stages of clinical development (Tables 2 and 3). SLPI is appears in both gene sets indicating its enhanced expression in DTCs as well as metastatic tumor.

**Table 2:** Gene transcripts differentially expressed in the metastasis of WHIM17 lineage mice relative to both control mice and primary xenograft tumor

Gene symbol	Gene name	Fold expression	*p* value	Targetable
*ALB*	Albumin	97.6	2.77E-08	
*IGF2*	Insulin-like growth factor 2	46.0	1.12E-06	
*FABP1*	Fatty acid binding protein 1	37.0	3.21E-07	
*GPC3*	Glypican 3	24.9	5.78E-09	Yes [38, 39]
*DCT*	Dopachrome tautomerase	21.3	2.15E-07	
*SPP1*	Secreted phosphoprotein 1	16.7	1.18E-07	
*FGG*	Fibrinogen gamma chain	16.5	2.56E-07	
*FGA*	Fibrinogen alpha chain	15.8	1.67E-07	
*MAL2*	Mal, T-cell differentiation protein 2	14.2	3.75E-08	
*APOB*	Apolipoprotein B	12.2	2.62E-07	
*CPE*	Carboxypeptidase E	10.7	1.11E-06	
*BAMBI*	BMP and activin membrane bound inhibitor	9.6	0.00021247	
*TYR*	Tyrosinase	9.4	3.52E-07	
*BCHE*	Butyrylcholinesterase	8.8	1.02E-06	
*CD24*	CD24 molecule	8.7	1.55E-05	
*LEF1*	Lymphoid enhancer binding factor 1	8.2	0.0001236	
*TSPAN8*	Tetraspanin 8	6.8	9.95E-06	
*SERPINA1*	Serpin family A member 1	6.2	2.00E-05	
*CGA*	Glycoprotein hormones, alpha polypeptide	6.2	7.70E-05	
*AZGP1*	Alpha-2-glycoprotein 1, zinc-binding	6.2	0.00126944	
*TFF1*	Trefoil factor 1	6.2	3.83E-05	
*HGD*	Homogentisate 1,2-dioxygenase	5.9	6.49E-05	
*TYRP1*	Tyrosinase related protein 1	5.2	2.68E-05	
*DSP*	Desmoplakin	5.1	8.91E-05	
*C3*	Complement C3	5.1	0.00067128	
*SLPI*	Secretory leukocyte peptidase inhibitor	5.1	7.24E-05

**Table 3:** Gene transcripts differentially expressed in the bone marrow of WHIM17 lineage mice relative to both control mice and primary xenograft tumor

Gene symbol	Gene name	Fold expression	*p* value	Targetable
*CD53*	CD53 molecule	4.1	0.0006062	
*PTPRC*	Protein tyrosine phosphatase, receptor type C	3.7	0.00474656	
*KIR3DL1*	Killer cell immunoglobulin like receptor, three Ig domains and long Cytoplasmic Tail 1	3.2	0.00145097	
*HBA1*	Hemoglobin subunit alpha 1	2.7	0.00117262	
*MT3*	Metallothionein 3	2.7	0.00033581	
*HBB*	Hemoglobin subunit beta	2.7	0.00086439	
*OSM*	Oncostatin M	2.5	0.00677886	
*MAP2K6*	Mitogen-activated protein kinase kinase 6	2.5	0.00632423	
*ARHGDIB*	Rho GDP dissociation inhibitor beta	2.4	0.00115363	
*SLPI*	Secretory leukocyte peptidase inhibitor	2.3	0.00071176	
*DDC*	Dopa Decarboxylase	2.3	0.00080507	Yes [40]
*SSTR4*	Somatostatin receptor 4	2.3	0.00058499	Yes [41, 42]
*MAPT*	Microtubule associated protein tau	2.3	0.0010915	
*HPR*	Haptoglobin-related protein	2.3	0.00139515	
*SPN*	Sialophorin	2.3	0.00092894	
*HLA-B*	Major histocompatibility complex, class I, B	2.2	4.27E-05	
*CISH*	Cytokine inducible SH2 containing protein	2.2	0.00021425	
*MST4*	Serine/threonine protein kinase 26	2.1	0.0035196	
*SERPINA5*	Serpin family A member 5	2.1	0.00622652	
*PTPN6*	Protein tyrosine phosphatase, non-receptor type 6	2.1	0.00735724	
*MMP17*	Matrix metallopeptidase 17	2.1	0.00493339	
*CEACAM6*	Carcinoembryonic antigen related cell adhesion molecule 6	2.0	0.00179388	

### The molecular profiles of PDX DTCs are also found in BM from patients with breast cancer

**Fig. 5:**
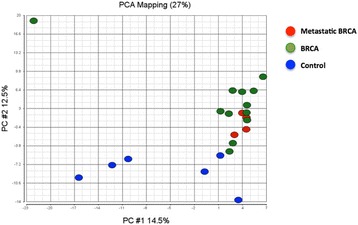
Principal component (PC) analysis (PCA) of 16 bone marrow (BM) samples from patients with breast cancer (12 without distant relapse and 4 with distant relapse) and 6 healthy female control BM samples, based upon a 420-gene signature identified from Washington University Human in Mouse (WHIM17) BM samples. *BRCA* breast cancer

Since gene expression patterns strongly suggested that cells in PDX mouse BM are derived from their primary xenograft tumor, and that there is a robust association between their presence and metastatic outcome, we next investigated whether gene expression in the BM of mouse PDX models could also be detected in the BM of patients with breast cancer, prior to the development of overt metastatic disease. Using previously generated BM gene expression microarray data from a cohort of treatment-naïve, clinical stage II/III patients with breast cancer and healthy female controls [8], we examined the expression of 420 unique genes from the PDX BM data set to determine whether they could detect differences between these populations. From the original set of 1979 “BM-specific” transcripts identified in the xenograft model, we derived a set of 420 transcripts that both could be mapped to human microarray expression data probe sets and that were annotated in PubMed citations with the key words “metastasis”, “invasion”, and “epithelial mesenchymal transition” (Fig. 1). Globally (Fig. 5) the WHIM17 BM gene set did not distinguish healthy female BM from BM of patients with breast cancer and had no ability to classify those patients who did or did not experience a distant metastatic event. However, we did identify a subset of 17 genes with expression that after correcting for false discovery, could distinguish between healthy BM and BM from patients with breast cancer (Table 4, Fig. 1). Given that the expression of these genes (1) is frequently associated with biological processes such as tumor cell proliferation, invasion, and metastasis; (2) are detectable only in BM from PDX mice that develop DTCs in their BM and distant organ metastases; and (3) are expressed in patients with breast cancer, as compared to healthy human BM, we propose that they are excellent biomarker candidates for future prospective studies to evaluate whether they can stratify patients with breast cancer for risk of recurrent disease based upon detection and classification of BM DTCs.

**Table 4:** WHIM bone marrow (BM) gene transcripts with expression that is statistically greater than background expression in normal human BM (*p* < 0.05, corrected for multiple comparisons using the false discovery (FDR) method)

Gene	Gene name	Drugs available against target gene
*CD163*	CD163 molecule	
*PDGFC*	Platelet derived growth factor C	
*GLIPR1*	GLI pathogenesis related 1	
*ANXA5*	Annexin A5	
*CPVL*	Carboxypeptidase, vitellogenic like	
*PF4*	Platelet factor 4	
*CYP1B1*	Cytochrome P450 family 1 subfamily B member 1	Yes [43]
*EPB41L3*	Erythrocyte membrane protein band 4.1 like 3	
*MEIS1*	Meis homeobox 1	
*TNFRSF17*	TNF receptor superfamily member 17	
*DLC1*	DLC1 rho GTPase activating protein	
*HEBP1*	Heme binding protein 1	
*CFDP1*	Craniofacial development protein 1	
*CD36*	CD36 molecule	
*CD33*	CD33 molecule	Gemtuzumab ozogamicin, acute myeloid leukemia [44–46]
*UGDH*	UDP-glucose 6-dehydrogenase	
*CD44*	CD44 molecule	

## Discussion

The presence of DTCs in the BM of patients with early-stage breast cancer identifies patients at high risk of recurrence [1]. Clinically, it is not clear whether the DTCs detected in the BM are the sole population of cells that later develop into metastatic foci, whether they represent the systemic burden of micrometastatic disease, or a combination of both. Regardless, clinical data demonstrate that DTCs can persist through chemotherapy and their presence after chemotherapy identifies a patient population at very high risk of recurrence, relative to those patients who clear their DTCs with chemotherapy [5, 27, 28]. In spite of these findings, DTC detection has not become a routine part of breast cancer patient management, primarily due to the limited number of targetable biomarkers and lack of a cost-effective, robust assay for detection which yields molecular information [8]. If the DTC phenotype is representative of the micrometastatic disease that will eventually become metastatic foci, then eliminating DTCs with targeted therapeutic approaches could prevent recurrent disease and result in a survival benefit to patients with breast cancer. Characterizing these cells is essential as our understanding of the parallel progression of the primary tumor and DTCs improves [6, 29].

To date, it has been difficult to study the role of DTCs in the metastatic cascade and to perform molecular characterization due to their rarity. However, several studies have shown that PDX models recapitulate the molecular phenotype and biological behavior, and are predictive of clinical response of primary human tumors [9–11, 30]. Several studies have characterized CTCs using PDX models in breast cancer to better understand the biology of this process [12, 13] and recently, DTCs have been reported using PDX models [14]. In the report by Giuliano et al. [14], DTCs were detected in 62% of PDX mouse BM.

In this study, we have focused on the combined use of PDX and patient BM samples to identify unique sets of gene transcripts that can both detect and classify breast cancer BM DTCs. A persistent limitation of this approach is the small number of stable PDX lines that can be created by implanting primary breast tumor tissues and, subsequently the number that demonstrate metastatic behavior. Of the five lines investigated in this study, only two (WHIM12 and WHIM17) developed solid organ metastases. Importantly, these were also the only two lines in which human-specific gene expression (presumably emanating from DTCs) could be detected in BM, strongly supporting the idea that DTC establishment is causal to or at least associated with distant organ metastasis in PDX mouse models. Furthermore, only one PDX line (WHIM17), derived from a patient with triple-negative breast cancer, consistently showed patterns of human gene expression that were reminiscent of a “mesenchymal-like” phenotype in multiple animals across multiple passages. It is curious that WHIM17 was the only tumor xenograft line to persistently propagate BM micrometastasis and it is recognized that the conclusions on gene expression biomarkers in human breast cancer BM samples may be necessarily constrained by this. Recently, Huang et al. [31] reported that later passages of the WHM17 tumor resembled a lymphoproliferative malignancy and not breast adenocarcinoma, based on RNA sequencing and phosphoproteomic studies. Such evolution of genomic features of PDX tumors when the tumor is propagated in mice has been reported recently [32]. Nevertheless, data from Li et al., who also performed molecular profiling of earlier passages of this tumor [11] similar to the specimens used in the current study, suggest that at least initially, WHIM17 is molecularly characteristic of other “basal-like” breast cancers.

Using the WHIM17 line, array-based gene expression profiling was performed to compare primary tumor tissue and a solitary metastatic lesion to multiple BM samples among different animals and different passages of the WHIM17 line. By using a human-specific, short-oligonucleotide array platform (i.e. Affymetrix GeneChips) and comparing WHIM17 BM samples with those of control mice, it was inferred that the majority of the 13,000+ transcripts identified (Additional file 3: Table S3) originated from human xenograft-derived tumor cells. Secondarily, by identifying those transcripts that were differentially expressed between the primary xenograft tumor and multiple WHIM17 BM samples, a list of candidate biomarkers of cells with high metastatic potential was created, which was further filtered and curated based upon association with published manuscripts related to metastasis biology (Fig. 1).

Among individual transcripts identified by this analysis were several genes known to be associated with the metastatic process (*ALCAM*, *MALAT1*) and EMT (*SNAI1*, *GSC*, *FOXC2*, *SIP1*) and breast cancer stem cells (*CD44*). Of equal importance, the absence of epithelial-specific transcripts was a conspicuous feature of these analyses. While expression of human epithelial genes such as cytokeratins in the BM have been the cornerstone for the identification of DTCs as tumor-derived cells, the absence of these genes is not surprising. Tumor cells undergo significant transformation in their morphology and molecular profiles during each stage of the metastatic process [33]. Only those tumor cells that successfully adapt to the unfamiliar molecular environments after being released from the primary tumor into circulation can survive and grow at a different anatomical location [34]. The BM environment has been shown to enhance this process through the action of various stromal cell populations [35]. Therefore, the loss of epithelial and breast-tissue-specific features in these cells can be attributed to the possible molecular transition which enables these cells to migrate to and survive in the BM matrix.

The clinical relevance of the gene expression patterns identified in WHIM mouse BM was evaluated in the BM of treatment-naïve patients with breast cancer as well. Although the xenograft used to create the WHIM17 line was a “triple negative” tumor, given the small number of BM gene expression data sets available, we considered BM samples from all patients, regardless of primary tumor molecular phenotype, as a single group. A small number of gene transcripts (Table 4) were differentially detected in patient BM as compared to healthy female controls, and the biological role and potential drug targetability of many of these genes (such as CD44, CD33, GLIPR1, and HEPB1) has been previously demonstrated. Although we were not able to determine the number of human DTCs present in the xenograft animals in the current study, previous studies have demonstrated that gene-expression-based detection of DTCs in human bone marrow samples using qRT-PCR can detect as few as 1 in 1 × 10^6 cells when analyzing 10^7 – 10^8 nucleated cells from a 3-mL BM aspirate, depending upon the specific gene transcript analyzed [36]. Whether one or more of these transcripts can be routinely detected above background expression in normal human BM, and whether expression of these gene(s) are actually prognostic for metastatic recurrence are questions currently being addressed. Although we were unable to analyze the expression of these genes in the peripheral blood of xenograft mice, it will also be interesting and clinically relevant to determine whether the expression of these genes can be detected in peripheral blood of patients with breast cancer, possibly providing a less invasive assay to predict early metastatic recurrence.

Our data would argue that DTCs derived from the primary tumor are mesenchymal-like and express genes associated with the metastatic process in animals and in humans. Presence of these cells was limited strictly to the BM of mice with metastatic tumor development and they were present in BM at a pre-metastatic time point. It has been suggested that the parallel progression of primary and metastatic tumors can occur simultaneously with early dissemination of cancer cells from the primary tumor [6, 7, 37]. Since the molecular features of the tumor cells remain consistent across passages, if DTCs were a general occurrence rather than associated with metastatic potential, we would expect to find DTCs in all animals from the same line irrespective of their metastatic outcome.

Our results support the use of the PDX mice as a clinically relevant model to examine the molecular features of DTCs, alteration over time, and whether elimination of BM DTCs using targeted therapies will result in abrogation of metastatic disease development.

## Conclusion

The presence of DTCs in the BM and its association with metastatic outcome was observed in the PDX model system. Our results provide an experimental support for the clinical association between DTCs in the BM of patients with early-stage breast cancer and recurrent disease development. We found that DTCs lose epithelial features and express genes associated with metastases and EMT. Moreover, using this system, we have identified new targetable genes associated with DTCs. Our data suggest that the PDX model provides a powerful tool to explore the metastatic process and the molecular characterization of DTCs.

## Additional files

Additional file 1: Table S1. Assay ids of the Taqman probes used in the study. (XLSX 9 kb)

Additional file 2: Table S2. qRT-PCR validation of the expression of six transcripts of genes previously implicated in tumorigenesis and metastasis and found to be elevated at least threefold in all seven WHIM BM samples by microarray analysis. (PPTX 33 kb)

Additional file 3: Table S3. Complete list of transcripts with significantly different expression between DTCs and primary tumors, and metastasis and primary tumor by microarray analysis. (XLSX 532 kb)

## Abbreviations

BM: Bone marrow
cDNA: Complementary DNA
C_T_: Cycle threshold
dC_T_: Delta C_T_
DMEM: Dulbecco’s modified Eagle’s medium
DTC: Disseminated tumor cells
EMT: Epithelial to mesenchymal transition
ER: Estrogen receptor
GAPDH: Glyceraldehyde-3-phosphate dehydrogenase
GFP: Green fluorescent protein
Her2: Human epidermal growth factor receptor 2
*hGAPDH*: human-specific GAPDH
PBS: Phosphate-buffered saline
PDX: Patient-derived xenograft
WHIM: Washington University Human in Mouse
