# Supplementary material for: Identifying biomarkers of breast cancer micrometastatic disease in bone marrow using a patient-derived xenograft mouse model
Source: Breast Cancer Res. 2018 Jan 2;20:2. doi: 10.1186/s13058-017-0927-1 (PMC5748947; doi:10.1186/s13058-017-0927-1)
Supplement: Supplementary file 2 — qRT-PCR validation of the expression of six transcripts of genes previously implicated in tumorigenesis and metastasis and found to be elevated at least threefold in all seven WHIM BM samples by microarray analysis. (PPTX 33 kb) [file 13058_2017_927_MOESM2_ESM.pptx]

## Slide 1
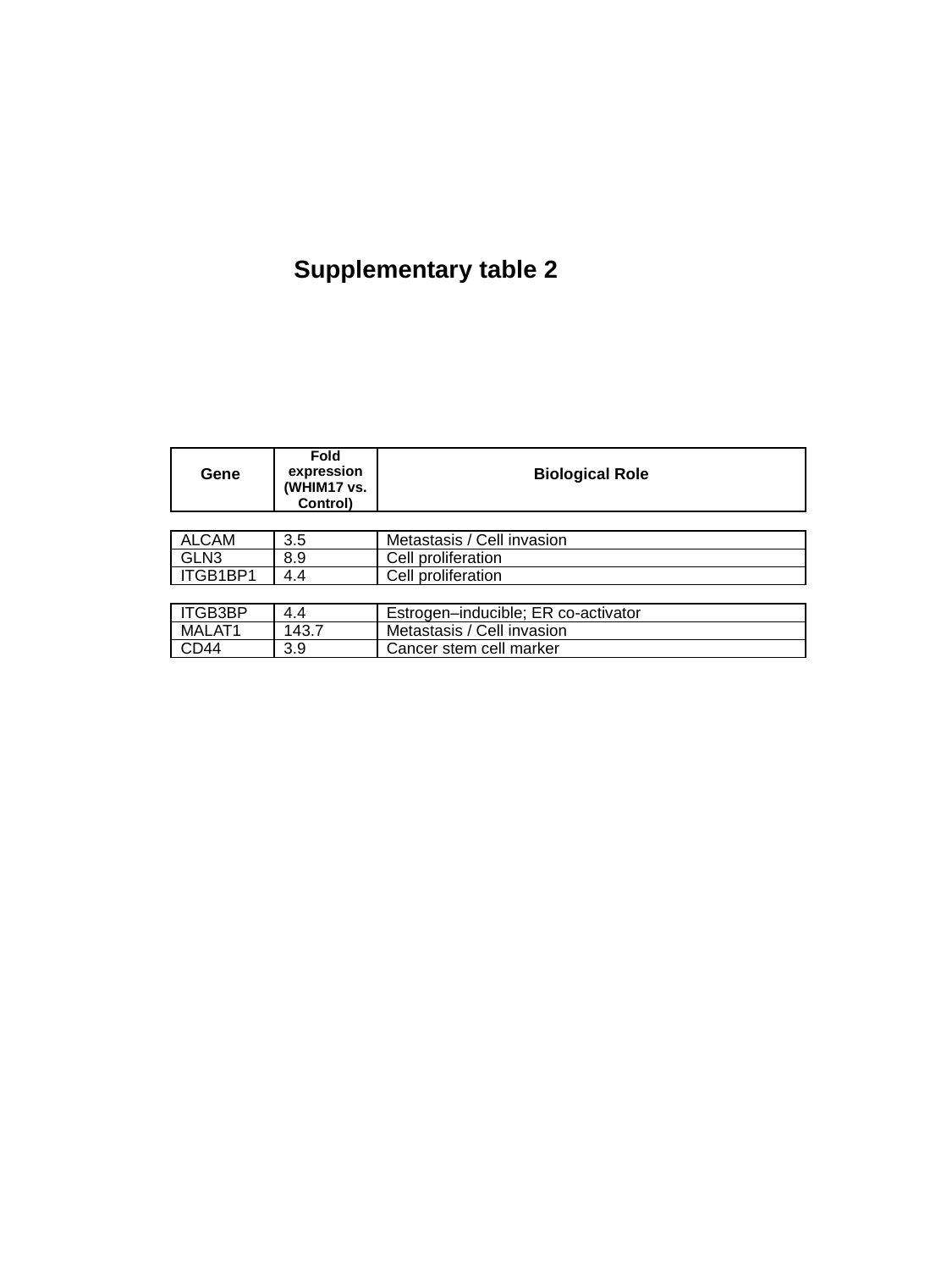

Supplementary table 2
| Gene | Fold expression (WHIM17 vs. Control) | Biological Role |
| --- | --- | --- |
| ALCAM | 3.5 | Metastasis / Cell invasion |
| --- | --- | --- |
| GLN3 | 8.9 | Cell proliferation |
| ITGB1BP1 | 4.4 | Cell proliferation |
| ITGB3BP | 4.4 | Estrogen–inducible; ER co-activator |
| --- | --- | --- |
| MALAT1 | 143.7 | Metastasis / Cell invasion |
| CD44 | 3.9 | Cancer stem cell marker |
